# Arbuscular mycorrhizal fungal communities in Jiang-flavor baijiu sorghum rhizosphere: environmental correlates and variation among production bases

**DOI:** 10.3389/fmicb.2026.1965730

**Published:** 2026-10-29

**Authors:** Peiyun Xie, Yanling Ren, Linlu Ye, Shuangshuang Hou, Yuejun He

**Affiliations:** 1 Guizhou Light Industry Polytechnic University, Guiyang, China; 2 Guizhou University, Guiyang, China

**Keywords:** arbuscular mycorrhizal fungi, environmental factors, Jiang-flavor baijiu, rhizosphere, waxy sorghum

## Abstract

Waxy sorghum (*Sorghum bicolor* var. glutinosum) is the principal raw material for Jiang-flavor baijiu, and its grain characteristics can vary across production environments with contrasting soil and climatic conditions. These environmental differences may also influence rhizosphere microbial communities that contribute to plant nutrient acquisition and crop performance. Among these microorganisms, arbuscular mycorrhizal fungi (AMF) are widespread root symbionts with important roles in nutrient acquisition and soil ecological processes. However, although previous studies of liquor-making sorghum have mainly focused on grain composition and bacterial communities, the spatial variation and environmental associations of rhizosphere AMF across major production areas remain poorly understood. This study investigated rhizosphere AMF communities in three major waxy sorghum production bases in Guizhou, China, using high-throughput 18S rDNA sequencing. Soil properties, grain quality attributes, and climatic data were analyzed via canonical correspondence analysis, LEfSe, and partial least squares path modeling. AMF alpha diversity did not differ significantly among the three production bases, whereas community composition showed clear site-associated differentiation, with *Paraglomus* representing the predominant classified genus in most samples. CCA indicated that AMF community variation was associated with multivariate edaphic, climatic, and topographic gradients; because several environmental variables were strongly collinear, these relationships were interpreted collectively rather than as independent effects of individual factors. Exploratory PLS-PM further indicated that the largest path associations linked the core AMF community with soil properties and soil properties with grain-quality indicators. Given the observational design with one production base and one weather station sampled per region, these relationships represent site-specific associations rather than evidence of causal environmental or microbial effects or region-wide patterns. Overall, these findings provide a baseline for understanding AMF community variation and soil-microbe-crop relationships at the three sampled Jiang-flavor baijiu sorghum production bases.

## 1 Introduction

Jiang-flavor baijiu depends on waxy sorghum (*Sorghum bicolor* var. glutinosum) as its principal raw material, and variation in grain composition can affect the quality characteristics of the resulting liquor (Zhang et al., 2022; Zhao et al., 2024). As sorghum production expands from long-established cultivation zones into newer production areas, maintaining stable raw-material quality becomes an important agronomic and industrial challenge. Regional differences in climate, topography, soil fertility, and land-use history can reshape the plant–soil interface, where microbial communities interact with crop nutrition and development. Previous work has documented regional variation in the physicochemical characteristics and bacterial communities associated with liquor-making sorghum (Xie et al., 2024). However, fungal symbionts at the plant–soil interface, particularly arbuscular mycorrhizal fungi (AMF), remain comparatively poorly characterized in this production system.

AMF form widespread symbiotic associations with plant roots and contribute to nutrient acquisition, particularly phosphorus uptake, under diverse soil conditions (Etesami et al., 2021; Wahab et al., 2023). Their ecological functions also extend to soil aggregation, carbon retention, plant growth, and stress responses, making them a central component of the rhizosphere microbiome (Rillig et al., 2001; Zhang et al., 2023). Evidence from several crops indicates that AMF can influence yield, grain composition, or nutritional quality, although the magnitude and direction of these effects depend on the host, soil environment, and management context (Zhang et al., 2017; Al Mutairi et al., 2020; Agnolucci et al., 2020). AMF communities are not spatially uniform. Their diversity and composition are shaped by climatic conditions, soil properties, host identity, agricultural management, and cultivation history. Global and regional studies have revealed strong environmental structuring of AMF distributions (Davison et al., 2015; Rodríguez Echeverría et al., 2017). Temperature and precipitation can influence AMF biogeography and community assembly, while soil nutrient status may alter plant dependence on mycorrhizal phosphorus acquisition (Wen et al., 2019; Sousa et al., 2022; Xu et al., 2022). Studies in vineyards and plantations further suggest that field age, soil traits, and management practices correspond to substantial shifts in AMF community composition (Liu et al., 2023; Noceto et al., 2023; Aguilar-Paredes et al., 2024; Catalani et al., 2024). Despite this progress, available studies of AMF in beverage-crop systems have largely examined vineyards. For liquor-making sorghum, existing research has emphasized grain composition, flavor-related raw-material properties, and bacterial communities (Zhang et al., 2022; Zhao et al., 2024; Xie et al., 2024; Niu et al., 2022). The ecological organization of rhizosphere AMF across contrasting sorghum-producing regions therefore remains comparatively underexplored.

Several unresolved issues limit an integrated understanding of the sorghum rhizosphere in Jiang-flavor baijiu production. First, it is unclear how strongly AMF assemblages differ between long-established and recently developed cultivation regions. Second, climatic, topographic, and edaphic variables often covary across production areas. Their joint associations with AMF community turnover have not been resolved within this specific cropping context. Consequently, the relative importance of factors such as temperature, precipitation, solar radiation, elevation, and soil nutrient status remains uncertain. A further gap concerns the connection between community structure and ecological function. Taxonomic differences alone do not establish whether regional AMF assemblages differ in predicted symbiotic roles or whether such variation is associated with soil conditions and sorghum quality attributes. Although AMF-mediated changes in crop quality have been reported in barley, rice, and other plant systems (Zhang et al., 2017; Al Mutairi et al., 2020), comparable evidence for waxy sorghum used in baijiu production is limited. An integrated framework is therefore needed to examine AMF diversity, environmental filtering, predicted functional traits, and quality-related variables without treating these components as separate processes. Such an approach may clarify how environmental and microbial dimensions should be considered together when developing future soil-management and microbial-resource strategies.

Against this background, we compared rhizosphere AMF communities at three representative waxy-sorghum production bases in Guizhou Province. We tested three hypotheses. First, we hypothesized that AMF community composition would differ among the three production bases, even if local alpha diversity remained relatively similar. Second, we hypothesized that variation in AMF community composition would be associated with edaphic and environmental gradients after accounting for collinearity among predictors. Third, we hypothesized that dominant and differentially represented AMF taxa would show site-associated distributions corresponding to local environmental conditions. To test these hypotheses, we combined AMF-targeted 18S rRNA gene sequencing with community-diversity analyses, differential-taxon analysis, and constrained environmental ordination. Soil properties and sorghum grain characteristics were additionally measured to characterize the agronomic environments represented by the three production bases. Because the study was observational and included one production base per region, associations between microbial, environmental, and crop variables were interpreted without inferring causality. By integrating community ecology with environmental and quality-related measurements, the study seeks to provide a regionally grounded basis for understanding AMF assembly in liquor-making sorghum systems.

## 2 Materials and methods

### 2.1 Study areas

Experimental samples were collected from three major production regions of waxy sorghum (*Sorghum bicolor* var. *glutinosum*) dedicated to Jiang-flavor baijiu, all located within the subtropical humid climate zone of Guizhou Province, China. The Renhuai (RH) region, a long-term cultivation area with more than 20 years of planting history, lies between 27°33′30″N–28°10′19″N and 105°59′49″E–106°35′50″E, with an average elevation of 400–600 m. This region provides highly favorable climatic conditions for sorghum growth. Jinsha County (JS), situated in northwestern Guizhou, extends from 27°07′N–27°46′N and 105°47′E–106°44′E, with an average elevation of 600–900 m. Since 2021, it has rapidly developed into a significant Jiang-flavor baijiu production base by leveraging its distinctive climatic and geographical advantages. Qixingguan District of Bijie (BJ), also in northwestern Guizhou, is located between 26°21′N–27°46′N and 103°36′E–106°43′E, with an average elevation reaching 1,500 m. Owing to its high altitude, this region experiences lower mean temperatures and greater diurnal variation. Since 2023, sorghum cultivation in BJ has become increasingly standardized, establishing the area as a key emerging node in the Guizhou baijiu industrial chain. The study area features a typical subtropical humid monsoon climate in karst mountainous areas. Under the unique karst geological conditions, the parent material of all three sampling sites is derived from carbonate rocks, and the predominant soil type is limestone soil. To explore the effects of different agricultural management practices on the rhizosphere microecology, typical field sites representing three local mainstream sorghum cultivation patterns were strategically selected in this study (Figure 1).

**Figure 1:**
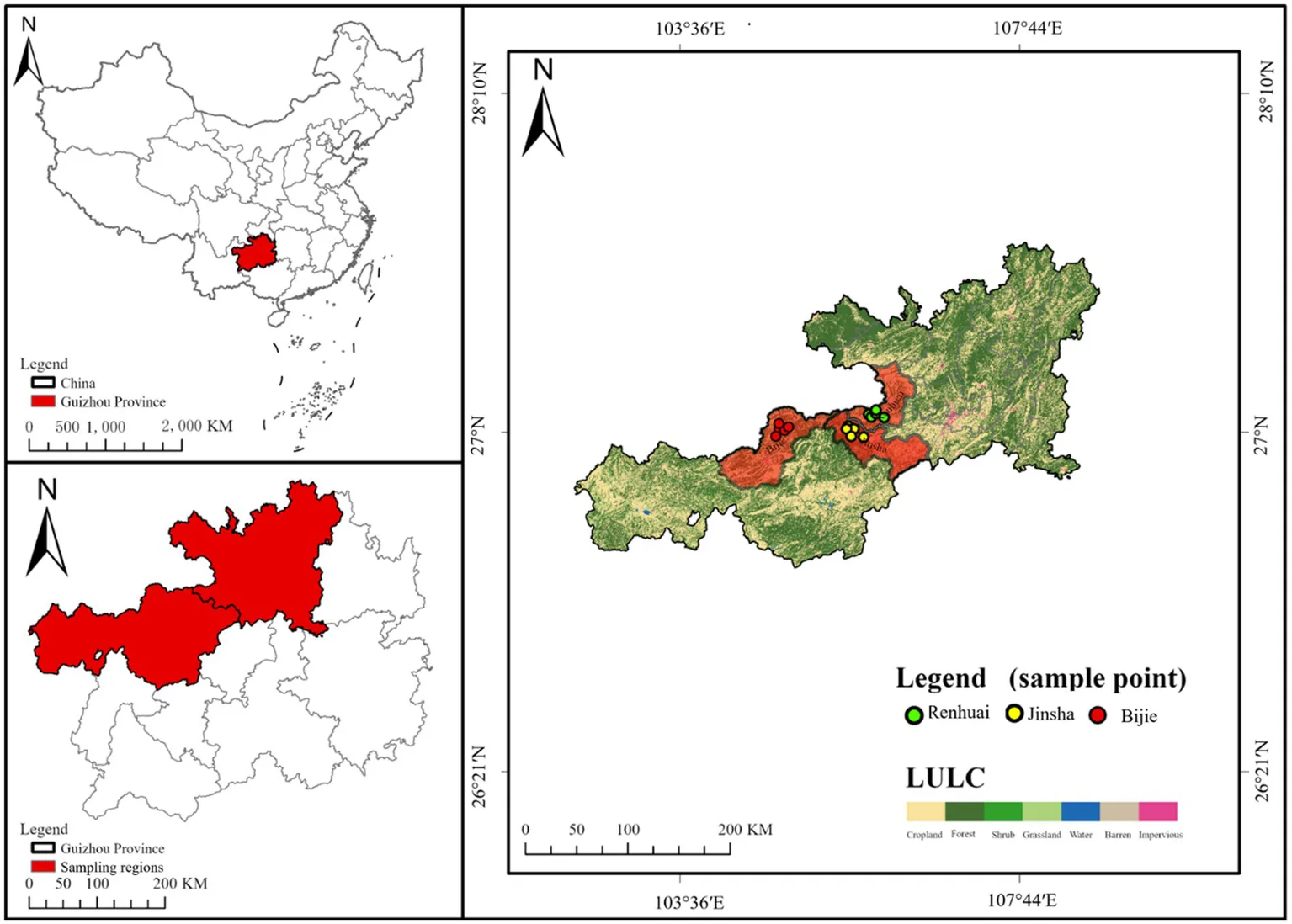
Geographic overview of the study region. The inset maps display the location of Guizhou Province within China and the scope of sampling regions within Guizhou. The main panel illustrates land- use and land- cover (LULC) and spatial distribution of sampling points for Bijie (red circles), Jinsha (yellow circles), and Renhuai (green circles). LULC categories: cropland, forest, shrub, grassland, water body, barren land, and impervious area.

### 2.2 Sample collection

In August 2024, soil samples were collected from waxy sorghum planting bases in RH, JS, and BJ, with precise geographic positioning recorded using a handheld GPS device. All selected plots were located on south-facing slopes and covered areas exceeding 45 mu. Field surveys confirmed the absence of interference from adjacent crops or vegetation. Interviews with local farmers verified that the selected plots had consistent planting dates, similar irrigation frequencies, and uniform pest control practices, thereby minimizing potential confounding factors. Within each production base, four spatially separated subplots (20 m × 20 m) were randomly established, with a minimum distance of 50 m between subplots. Each subplot constituted one field sampling unit. Within each subplot, 15 healthy sorghum plants of similar growth status were selected using an S-shaped sampling strategy. Rhizosphere soils collected from the 15 plants were homogenized to generate one composite sample. Thus, four composite rhizosphere-soil samples were obtained from each production base, yielding 12 samples in total. The individual plants were treated as subsamples for generating each composite sample and were not analyzed as independent replicates. Because only one production base was sampled within each geographic region, the study does not provide independent replication of the regional factor; consequently, comparisons are interpreted as contrasts among the three sampled production bases. All processed samples were immediately sealed in sterile polyethylene self-sealing bags, quickly frozen in portable refrigerators filled with dry ice, and promptly transported back to the laboratory for subsequent high-throughput sequencing and physicochemical property analysis.

### 2.3 Chemical analysis and climate data

Soil samples were analyzed using agricultural chemical analysis of soil protocols for the indicators, organic matter, total nitrogen, total phosphorus, total potassium (Bao, 2000). The waxy sorghum crops at the three experimental bases [A (RH), B (JS), and C (BJ)] were planted in March and harvested in September 2024, spanning a full growth cycle from spring to autumn. Rhizosphere soil sampling was strategically conducted in August, timed to coincide with the grain-filling stage when microbial interactions and nutrient allocation to grains are most active. Meteorological data during the growth cycle—including average monthly temperature, precipitation, relative humidity, average wind speed, net solar radiation, and average sunshine hours—were obtained from the daily value dataset of surface climate data provided by the China Meteorological Science Data Sharing Service Network,^1^ using records from Renhuai Station (Station ID: 57710), Jinsha Station (Station ID: 57714), and Bijie/Qixingguan Station (Station ID: 57707). Starch content in sorghum was measured following the method of McCleary et al. (1994). Amylose and amylopectin contents were determined according to ISO 6647-2:2007, protein content according to ISO 11085:2015, fat content according to ISO 20483:2013, and tannin content according to ISO 9648:1988. Grain yield of waxy sorghum was determined at the physiological maturity stage in September 2024 for each experimental subplot. To eliminate marginal effects, a standardized 10 m × 10 m sampling area was designated for yield measurement in each replicate. All sorghum plants within the quadrat were manually harvested and threshed. Grains were air-dried to a constant weight to remove residual field moisture and weighed using an electronic analytical balance. The measured grain weight was converted to unit area yield (kg·hm^−2^).

### 2.4 DNA extraction, PCR amplification, and high-throughput sequencing

Total genomic DNA from soil samples was extracted following the manufacturer’s instructions of the Soil DNA Kit (Omega Bio-tek, Norcross, GA, USA). The quality of extracted DNA was assessed via 1% agarose gel electrophoresis, while its concentration and purity were determined using a NanoDrop 2000 spectrophotometer (Thermo Scientific, USA). Nested PCR amplification was performed using two pairs of specific primers targeting AMF: AML1/AML2 (5′-ATCAACTTTCGATGGTAGGATAGA-3′; 5′-GAACCCAAACACTTTGGTTTCC-3′) and AMV4-5NF/AMDGR (5′-AGCTCGTAGTTGAATTTCG-3′; 5′-CCCAA-CTATCCCTATTAATCAT-3′) (Yang et al., 2022). The PCR reaction mixture (20 μL total volume) consisted of 4 μL 5 × TransStart FastPfu Buffer, 2 μL 2.5 mmol/L dNTPs, 0.8 μL each of forward and reverse primers (5 μmol/L), 0.4 μL TransStart FastPfu DNA Polymerase, 10 μL DNA template, and nuclease-free water to adjust the volume. The amplification protocol was as follows: initial denaturation at 95 °C for 3 min; 27 cycles of denaturation at 95 °C for 30 s, annealing at 55 °C for 30 s, and extension at 72 °C for 45 s; final extension at 72 °C for 10 min; and holding at 4 °C. Each sample was amplified in triplicate to ensure reproducibility. Amplicons from the triplicate reactions of each sample were pooled, and target bands were recovered via 2% agarose gel electrophoresis. Purification of the recovered products was performed using the AxyPrep DNA Gel Extraction Kit (Axygen Biosciences, Union City, CA, USA). The purity of purified amplicons was verified by 2% agarose gel electrophoresis, and their concentrations were quantified using a Quantus™ Fluorometer (Promega, USA) (Bokulich et al., 2013). Finally, libraries were constructed from the purified PCR products using the NEXTFLEX Rapid DNA-Seq Kit, and high-throughput sequencing was conducted on the Illumina MiSeq platform by a commercial biotechnology company (Novogene Co., Ltd., Beijing, China).

### 2.5 Data statistical analysis

Paired-end reads generated by Illumina sequencing were merged using FLASH (Santos-González et al., 2007), and primer sequences were trimmed. For AMF community analysis, the sequences were amplified using AMF-specific nested primers targeting the 18S rDNA region, and quality filtering, denoising, and amplicon sequence variant (ASV) inference were performed using the DADA2 plugin in QIIME2 (v.2024.2) (Ma et al., 2019). Taxonomic assignment of AMF ASVs was conducted against the MaarjAM database (Noceto et al., 2023). All 12 composite rhizosphere-soil samples were included in the AMF community analyses.

Soil physicochemical properties, grain yield, and grain-quality traits were summarized as mean ± standard deviation for each sampled production base. Differences among Renhuai, Jinsha, and Bijie were evaluated using one-way analysis of variance (ANOVA). Statistical significance was defined as *p* < 0.05. These analyses were used to evaluate differences in soil properties, grain yield, and grain composition among the three sampled production bases.

AMF community composition was summarized at the genus level using relative abundance. Differentially represented taxa among production bases were explored using linear discriminant analysis effect size (LEfSe), with a significance threshold of *p* < 0.05 and an LDA score threshold of 2.0. Because of the limited sample size, LEfSe results were interpreted as exploratory differences in taxon representation rather than as definitive regional biomarkers.

Alpha diversity of the AMF communities was evaluated using Shannon diversity, Pielou’s evenness, Simpson, and dominance indices. Differences in alpha-diversity indices among production bases were assessed using Kruskal-Wallis test with Dunn’s post-hoc test, with *p* < 0.05 considered statistically significant. Beta diversity was evaluated primarily using Bray–Curtis dissimilarities, followed by principal-coordinate analysis (PCoA) to visualize variation in AMF community composition among samples. Differences in community composition among the three sampled production bases were further evaluated using the multi-response permutation procedure (MRPP).

Associations between AMF community composition and environmental variables were evaluated using canonical correspondence analysis (CCA). Before interpretation of the constrained ordination, multicollinearity among the 12 candidate environmental variables was assessed using pairwise correlation analysis followed by iterative variance inflation factor (VIF) screening.

Community analyses were performed using the NovoMagic Cloud Platform (Novogene Co., Ltd., Beijing, China). Statistical analyses and visualization were additionally conducted in R (v.4.0.3), using packages including *ggplot2* and *phyloseq* (Xu et al., 2022; Brands et al., 2018).

## 3 Results

### 3.1 Variation among production bases in soil properties, grain yield, and sorghum grain composition

Soil properties differed among the three sampled production bases (Supplementary Table S1). RH had the highest SOM and TP, JS had the highest TN, and BJ had the highest TK. Grain yield and composition also varied among production bases (Supplementary Table S2; Figure 2). JS showed the highest grain yield, RH had the highest total starch and amylopectin contents, whereas BJ showed comparatively higher lipid and protein contents. Tannin content was lower in JS than in RH and BJ.

**Figure 2:**
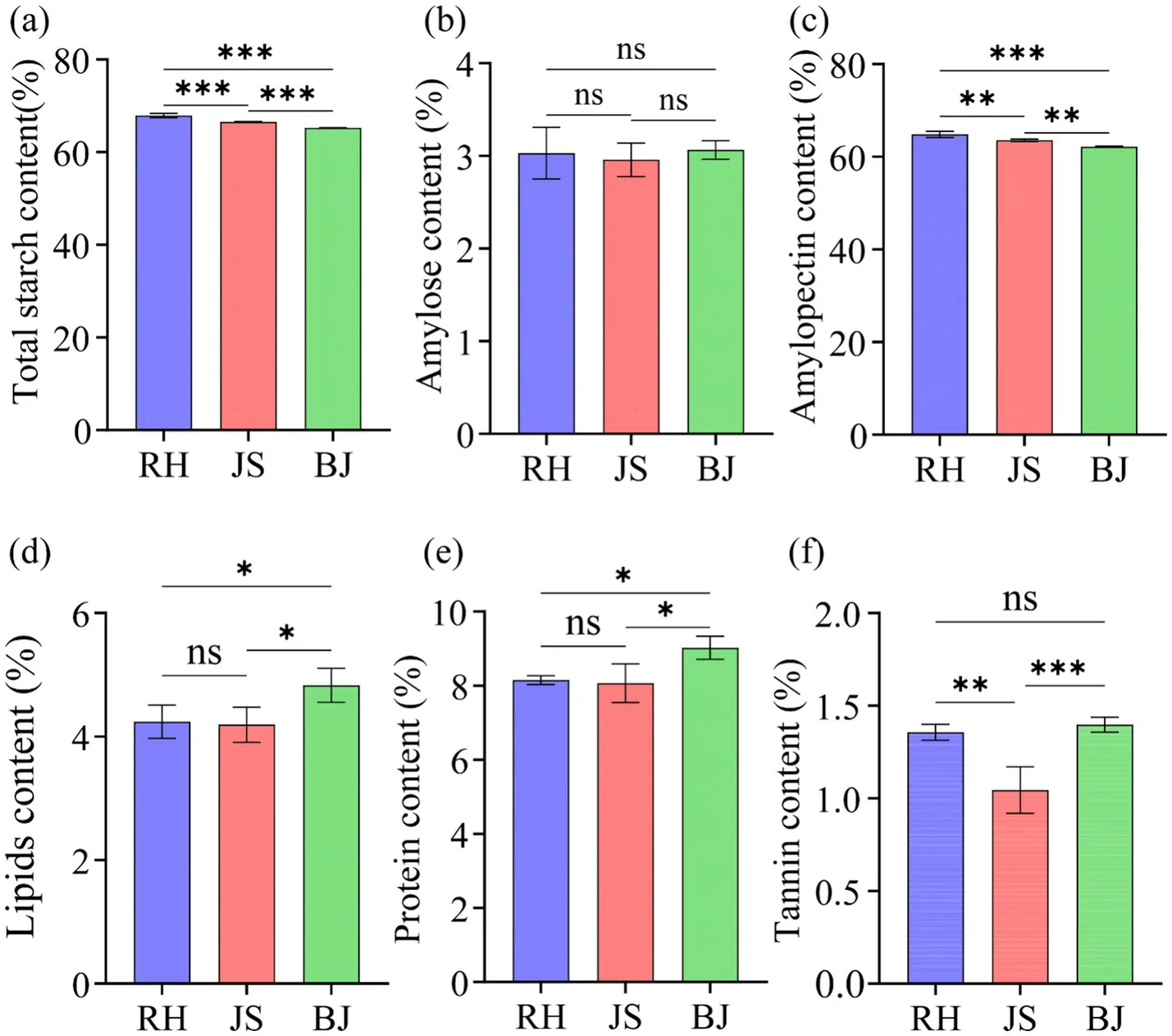
Comparative analysis of physicochemical properties of sorghum grains from the three sampled production bases. Panels **(a–f)** show total starch content, amylose content, amylopectin content, lipid content, protein content, and tannin content, respectively. RH, JS, and BJ denote sorghum grain samples collected from the three sampled production bases. Ns indicates no significant difference; *, **, and *** indicate significant differences at the levels of *p* < 0.05, *p* < 0.01, and *p* < 0.001, respectively.

### 3.2 AMF community composition across the sampled production bases

The Shannon rarefaction curves increased sharply at low sequence numbers and subsequently approached stable plateaus in all 12 samples (Supplementary Figure S1). At the genus level, *Paraglomus*, *Glomus*, and the pooled “Others” category together accounted for approximately 97–100% of each community profile (Figure 3). Among the named genera, *Paraglomus* had the highest relative abundance in every sample. In RH (A1–A4), “Others” constituted the largest overall fraction, ranging from 48 to 87%, whereas *Paraglomus* and *Glomus* ranged from 8 to 34% and from 5 to 18%, respectively. A3 contained the lowest proportion of *Paraglomus* (8%) and the highest proportion of “Others” (87%) among all samples. Within JS (B1–B4), *Paraglomus* was the largest category in B1 and B3, accounting for 50 and 64%, respectively, whereas “Others” was the largest category in B2 and B4, accounting for 54% in both samples. The relative abundance of *Glomus* in Jinsha ranged from 11 to 15%. In BJ (C1–C4), *Paraglomus* was the largest category in all four samples, ranging from 44% in C3 to 77% in C4; the corresponding proportions of “Others” and *Glomus* ranged from 17 to 40% and from 5 to 15%, respectively. The remaining genera—*Claroideoglomus*, *Gigaspora*, *Diversispora*, *Ambispora*, *Acaulospora*, *Archaeospora*, and *Scutellospora*—collectively contributed no more than approximately 3% to any individual sample. LEfSe analysis highlighted the class Glomeromycetes for group A in the A–B comparison (Figure 4a). In the A–C comparison, Glomeromycetes was again associated with group A, whereas the family Paraglomeraceae, order Paraglomerales, and class Paraglomeromycetes were associated with group C (Figure 4b). No significant differential taxa were detected between groups B and C.

**Figure 3:**
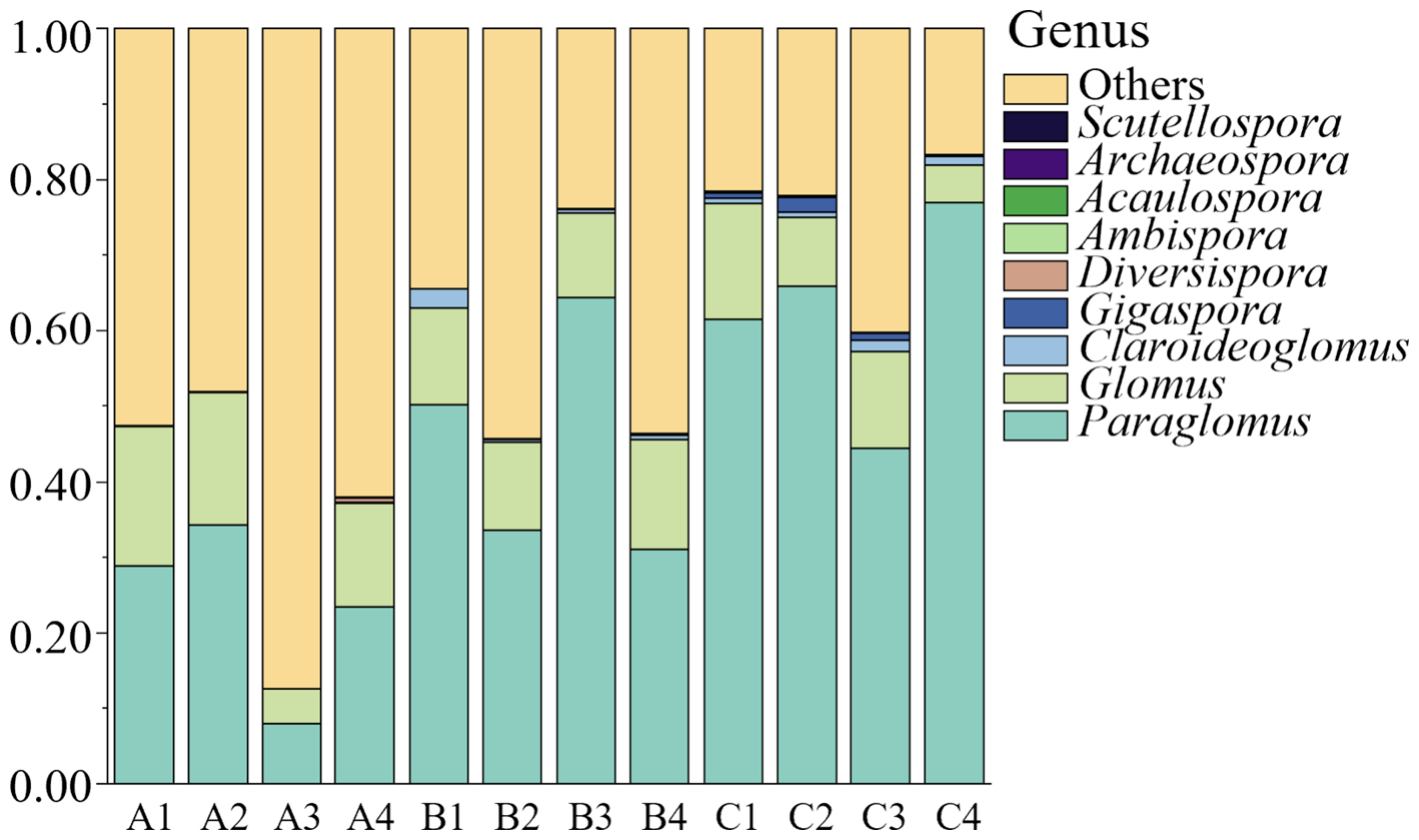
Stacked bar plot illustrating the relative abundance of arbuscular mycorrhizal fungi at the genus level. Samples from RH (A1–A4), JS (B1–B4), and BJ (C1–C4) are shown along the *x*-axis. Genus categories are distinguished by different colours as listed in the legend. “Others” represents AMF sequences not assigned to the individually displayed genera, including genus-level unclassified sequences.

**Figure 4:**
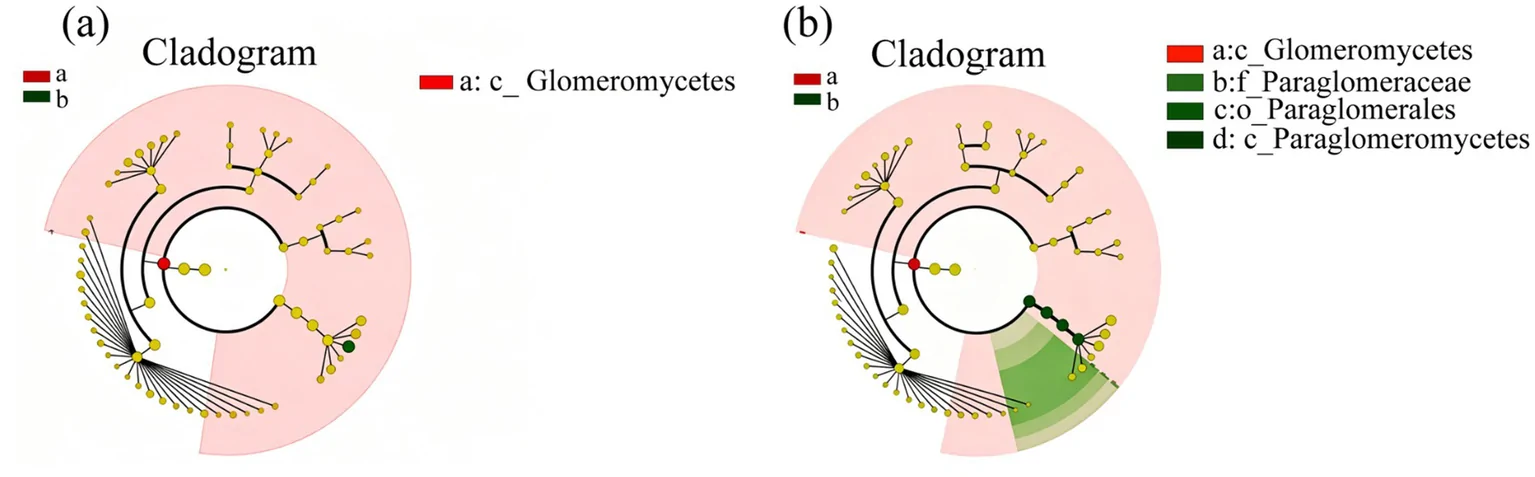
Differences in soil arbuscular mycorrhizal fungal taxa among the three sampled production bases based on LEfSe analysis. **(a)** Comparison between group A and group B; **(b)** Comparison between group A and group C. No significant differential taxa were detected in the comparison between group B and group C.

### 3.3 Variations in arbuscular mycorrhizal fungal diversity

The alpha-diversity indices were comparable among RH (A), JS (B), and BJ (C) (Figure 5). The distributions of Pielou’s evenness, Shannon, dominance, and Simpson indices overlapped across the three sampled production bases, and no significant differences were detected in any pairwise comparison (all *p* > 0.05).

**Figure 5:**
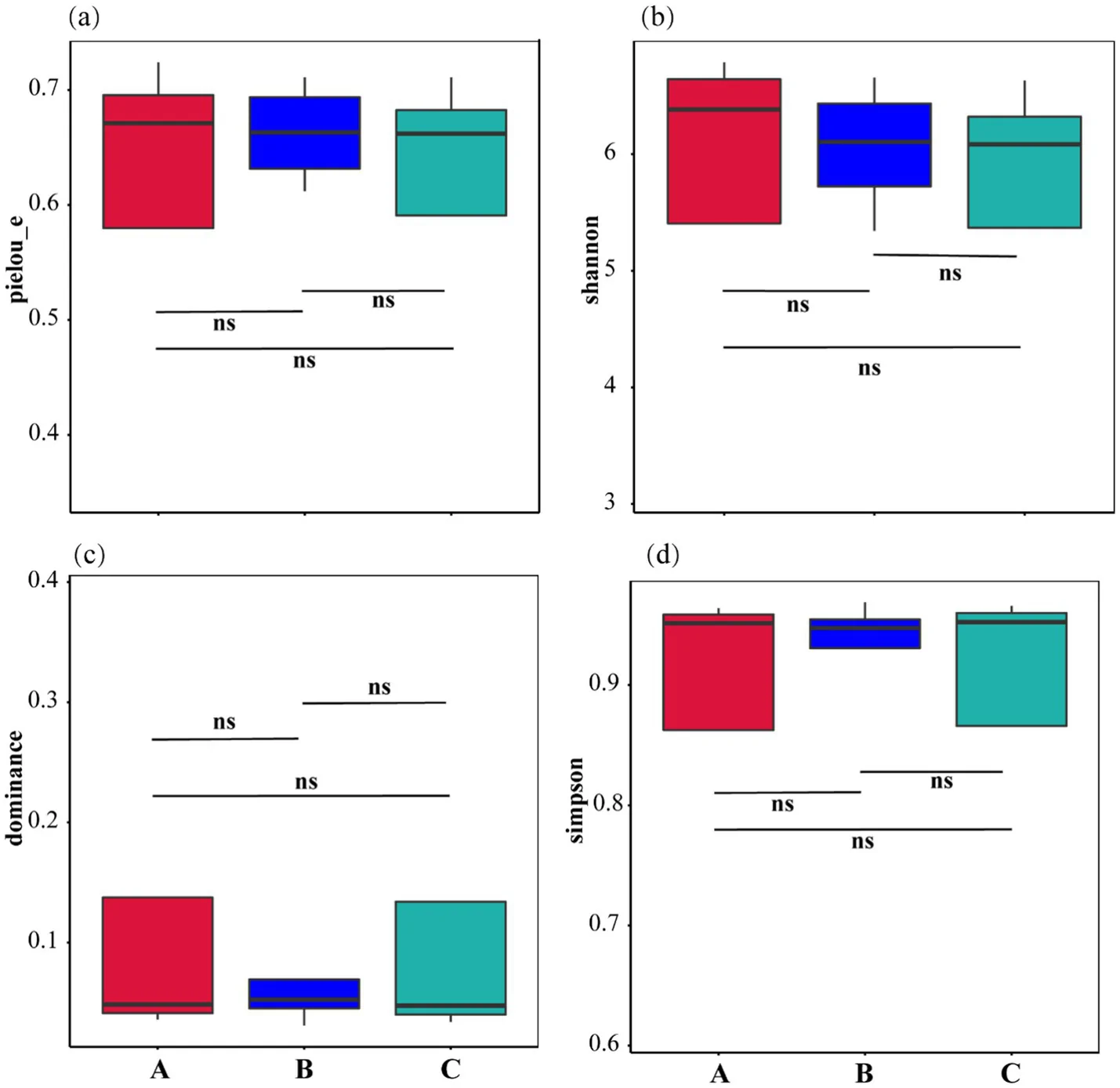
Alpha diversity indices of three groups. Boxplots show median, interquartile range and 1.5 × IQR whiskers. A: RH; B: JS; C: BJ. Significance was evaluated by Kruskal-Wallis test with Dunn’s *post-hoc* test (*p* < 0.05); *ns* = not significant. Panels **(a–d)** represent Pielou evenness, Shannon, Dominance and Simpson indices, respectively.

Thus, none of the four alpha-diversity metrics showed statistically significant variation among the three sampled production bases. In contrast, Bray–Curtis-based principal-coordinate analysis revealed clear site-associated differentiation in AMF community composition among the three bases (Figure 6). PC1 and PC2 explained 36.82 and 14.67% of the total variation, respectively, accounting for 51.49% cumulatively. RH samples clustered at negative PC1 scores, whereas JS and BJ samples were distributed at positive PC1 scores. Along PC2, Jinsha samples were located at positive scores, while Bijie samples were primarily positioned at negative scores. The 95% confidence ellipses occupied separate regions of the ordination space, and the Bijie samples showed a broader distribution than the Renhuai and Jinsha samples. Consistent with the PCoA pattern, MRPP detected significant differences in AMF community composition for all pairwise base comparisons (Table 1). The Renhuai–Jinsha comparison had an MRPP A value of 0.24, with an observed delta of 0.59, an expected delta of 0.77, and a significance value of 0.02. The Renhuai–Bijie comparison yielded A = 0.21, observed delta = 0.63, expected delta = 0.79, and *p* = 0.03, whereas the Jinsha–Bijie comparison yielded A = 0.09, observed delta = 0.62, expected delta = 0.69, and *p* = 0.03. Among the three comparisons, the largest MRPP A value occurred between Renhuai and Jinsha, while the smallest occurred between Jinsha and Bijie.

**Figure 6:**
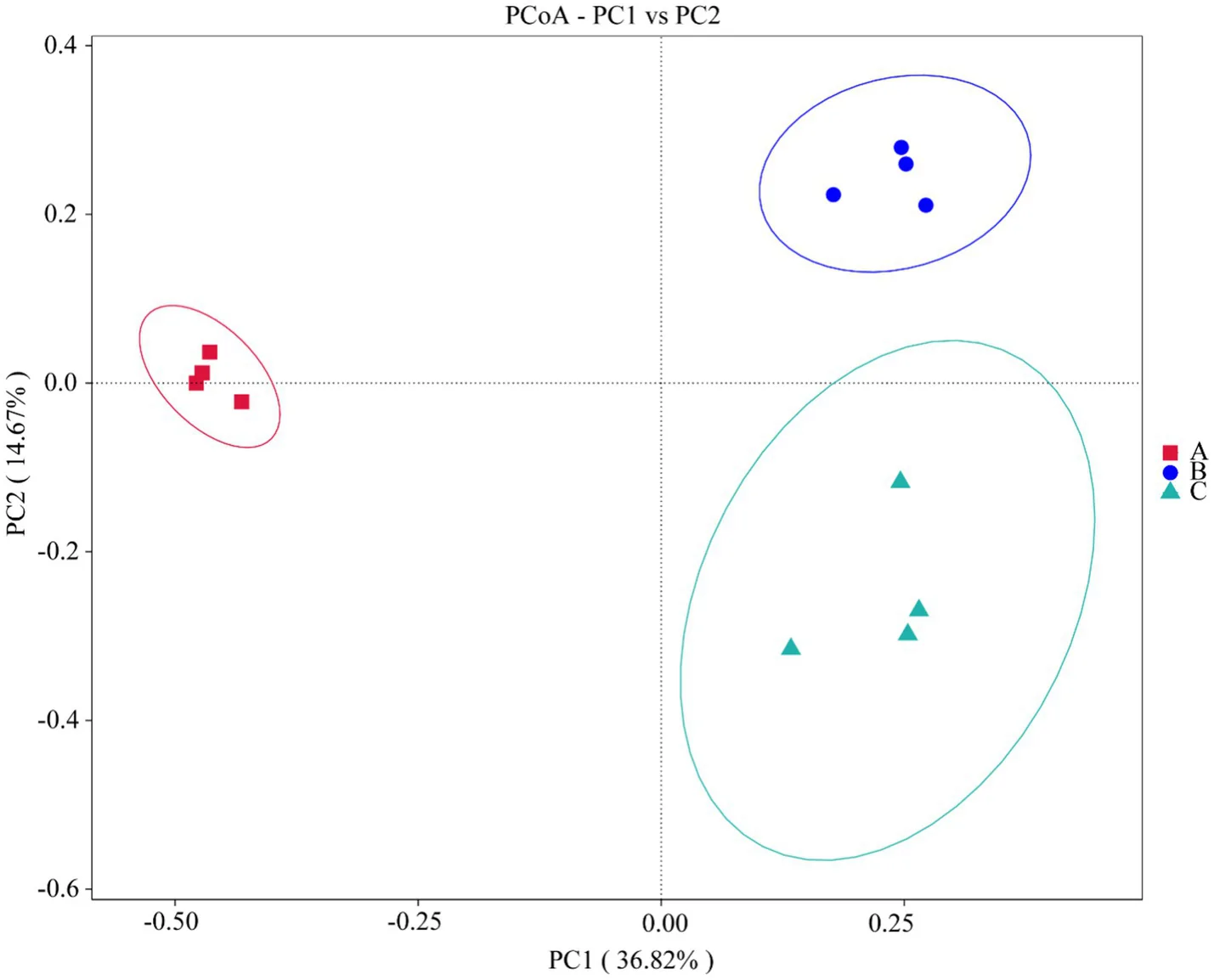
Principal-coordinate analysis (PCoA) based on Bray-Curtis distance illustrating the differences in arbuscular mycorrhizal fungal (AMF) community composition among the three sampled production bases. A, Renhuai; B, Jinsha; C, Bijie. PC1 and PC2 explained 36.82 and 14.67% of the total variation, respectively. Ellipses denote 95% confidence intervals for each group.

**Table 1:** Multi-response permutation procedure (MRPP) analysis of AM fungal community differences among production bases.

Group	A	Observed-delta	Expected-delta	Significance
A–B	0.24	0.59	0.77	0.02
A–C	0.21	0.63	0.79	0.03
B–C	0.09	0.62	0.69	0.03

A smaller observe-delta value indicates lower within-group variation, whereas a larger expect-delta value indicates greater among-group variation. An A-value > 0 suggests that among-group variation exceeds within-group variation, while an A-value < 0 indicates that within-group variation is greater than among-group variation. A Significance value < 0.05 denotes statistically significant differences.

### 3.4 Environmental associations with AMF community distribution

Prior to interpretation of the constrained ordination, multicollinearity among the 12 environmental variables was evaluated using pairwise correlations and iterative variance inflation factor (VIF) screening (Supplementary Table S3). The screening indicated substantial redundancy among several environmental variables. TK showed the strongest initial redundancy and was removed first during the screening procedure, followed sequentially by ELE (VIF = 12,975.5), NSR (VIF = 880.9), TN (VIF = 240.7), AWS (VIF = 50.1), and RH (VIF = 21.8). The remaining variables, AMT, Pr, ASH, Slo, SOM, and TP, had final VIF values below 10. These diagnostics were used to identify groups of strongly covarying environmental variables and to avoid interpreting individual CCA vectors as independent environmental effects. All measured environmental variables are shown in Figure 7 to illustrate the overall environmental gradients represented by the three production bases, whereas highly collinear variables are interpreted collectively rather than as independent predictors.

**Figure 7:**
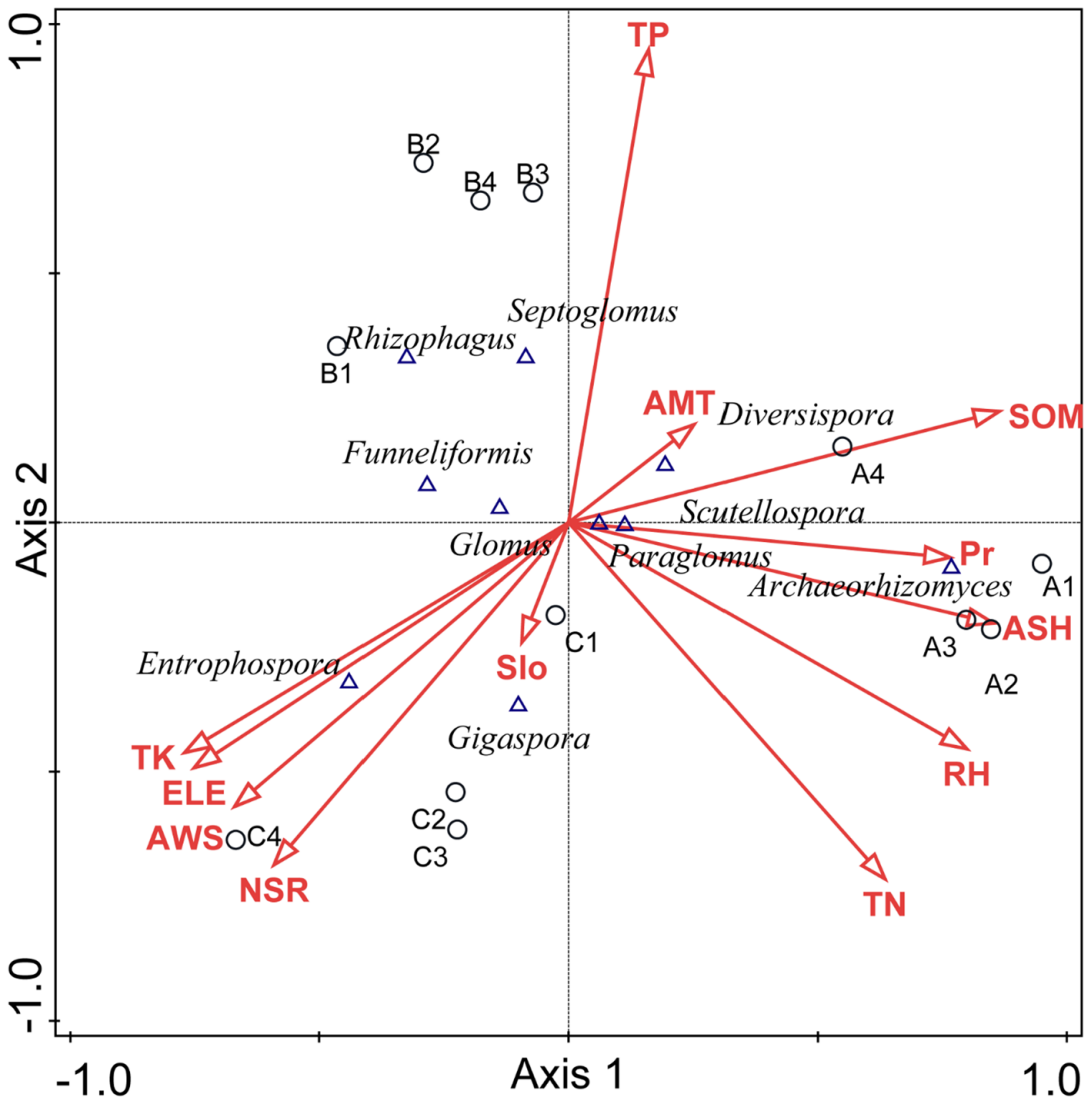
Canonical correspondence analysis (CCA) of AMF community composition and environmental variables across the three sampled production bases. Red arrows represent environmental gradients and blue triangles represent AMF genera. Environmental variables identified as highly collinear during VIF screening are displayed to illustrate their covariance structure but are not interpreted as independent environmental effects. Circles represent field samples collected from Renhuai (A1–A4), Jinsha (B1–B4), and Bijie (C1–C4).

Canonical correspondence analysis revealed clear site-associated differentiation of the AMF communities along the environmental gradients (Figure 7). The eigenvalues of CCA axes 1–4 were 0.35, 0.31, 0.15, and 0.09, respectively (Table 2). Axis 1 accounted for 35.57% of the explained variation, and the first two axes together accounted for 66.10%. The cumulative explained variation increased to 81.15% for the first three axes and 90.72% for all four axes. The corresponding pseudo-canonical correlations were 0.95, 0.96, 0.95, and 0.96.

**Table 2:** Eigenvalues and cumulative explained variation of the first four axes in canonical correspondence analysis (CCA) ordination.

Statistic	Axis 1	Axis 2	Axis 3	Axis 4
Eigenvalues	0.35	0.31	0.15	0.09
Explained variation (cumulative)	35.57	66.1	81.15	90.72
Pseudo-canonical correlation	0.95	0.96	0.95	0.96

Pseudo-canonical correlation represents the correlation between species scores and environmental factor scores for each CCA axis.

The first two CCA axes separated the samples according to production base. RH samples were distributed on the positive side of Axis 1, with A4 in the upper-right quadrant and A1–A3 in the lower-right quadrant. Jinsha samples occurred on the negative side of Axis 1 and the positive side of Axis 2, whereas BJ samples were predominantly located in the lower-left quadrant; C1 was positioned close to the ordination origin. The vectors for soil organic matter (SOM) and average monthly temperature (AMT) extended toward the upper-right quadrant, where A4 was located. In contrast, precipitation (Pr), average sunshine hours (ASH), relative humidity (RH), and total nitrogen (TN) were oriented toward the positive side of Axis 1 and the negative side of Axis 2, corresponding to the sector occupied by A1–A3. Total phosphorus (TP) was directed predominantly along the positive side of Axis 2, while total potassium (TK), elevation (ELE), average wind speed (AWS), and net solar radiation (NSR) extended toward the lower-left quadrant occupied by C2–C4. The slope vector was comparatively close to the origin and pointed toward the lower-left quadrant near C1.

Several environmental variables identified as highly collinear during VIF screening showed similar orientations in the ordination. In particular, TK, ELE, AWS, and NSR were closely aligned toward the lower-left quadrant occupied by C2–C4, indicating that these variables represented a strongly covarying environmental gradient rather than independent environmental effects. RH and TN were oriented toward the lower-right sector of the ordination. Accordingly, the directions of these highly collinear vectors were interpreted as components of broader site-associated environmental gradients rather than as evidence that any single variable independently structured the AMF community.

The distributions of AMF genera also differed across the ordination space. *Diversispora* was located in the upper-right quadrant in the directions of the SOM and AMT vectors. Scutellospora and *Paraglomus* were positioned close to the origin on the positive side of Axis 1, whereas *Archaeorhizomyces* occurred in the lower-right quadrant. Rhizophagus, Septoglomus, and *Funneliformis* were distributed in the upper-left quadrant together with the Jinsha samples. By comparison, *Entrophospora* and *Gigaspora* occurred in the lower-left quadrant, where the BJ samples and the TK, ELE, AWS, and NSR vectors were located. *Glomus* was positioned close to the center of the ordination. These spatial relationships indicate covariation between AMF community composition and environmental gradients but should not be interpreted as independent or causal effects of individual environmental variables. Because the three production bases each represent a single geographic location with corresponding site-specific climate data, the CCA ordination should be interpreted as a descriptive comparison among three sites rather than as evidence of independent regional environmental effects. The observed vector directions indicate covariance at the site level and cannot be generalized to other locations.

### 3.5 PLS-PM analysis of associations among climate, AMF, soil properties, and grain quality indicators

The partial least-squares path model showed three statistically significant paths among the latent variables (Figure 8). The core AMF community had a significant positive relationship with soil properties, with a standardized path coefficient of 0.7263 (*p* < 0.05). Soil properties were positively related to grain-quality indicators, and this path had the largest positive coefficient in the model (0.8063, *p* < 0.001). In contrast, soil properties were significantly negatively related to grain yield, with a coefficient of −0.6771 (*p* < 0.05). The model also showed negative coefficients from climate to the core AMF community (−0.3622), from climate to soil properties (−0.092), and from the core AMF community to grain yield (−0.1746), together with a positive coefficient from the core AMF community to quality indicators (0.2042); these paths were not accompanied by significance symbols.

**Figure 8:**
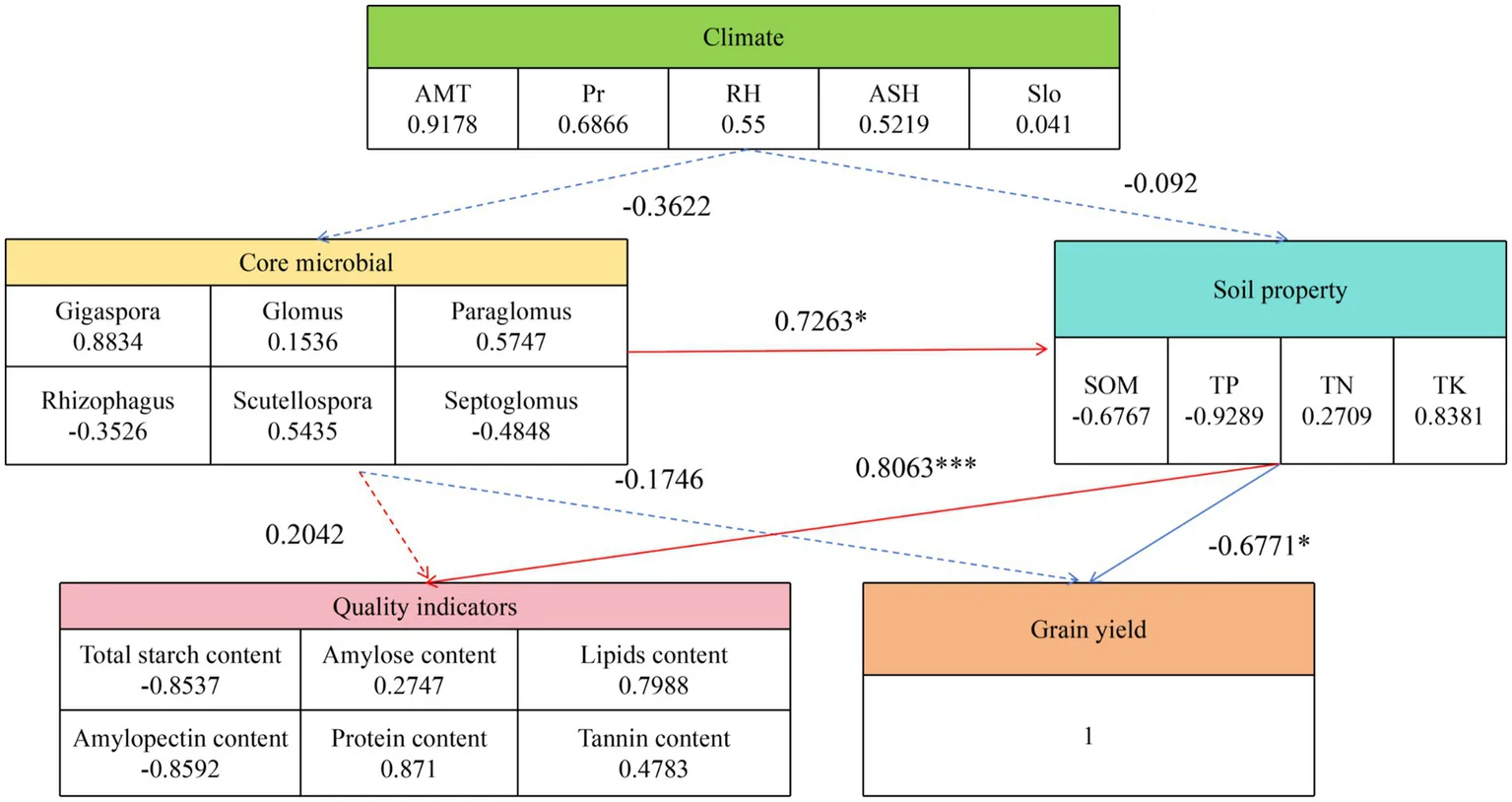
Partial least squares path model (PLS-PM) illustrating the path associations among climate factors, core arbuscular mycorrhizal fungal communities, soil properties, sorghum grain yield, and grain quality indicators. Red solid arrows represent significant positive path coefficients; blue dashed arrows represent negative path coefficients. Asterisks indicate statistical significance: *p* < 0.05, **p < 0.001. Values adjacent to arrows are standardized path coefficients. Values within each latent variable box are the loading coefficients of corresponding manifest variables. This exploratory model describes associations among the three sampled bases.

Within the climate construct, average monthly temperature had the largest loading coefficient (0.9178), followed by precipitation (0.6866), relative humidity (0.5500), average sunshine hours (0.5219), and slope (0.0410). The core AMF community construct was positively loaded by *Gigaspora* (0.8834), *Paraglomus* (0.5747), *Scutellospora* (0.5435), and *Glomus* (0.1536), whereas *Rhizophagus* and *Septoglomus* had negative loadings of −0.3526 and −0.4848, respectively. For the soil-property construct, total potassium and total nitrogen had positive loadings of 0.8381 and 0.2709, whereas total phosphorus and soil organic matter had negative loadings of −0.9289 and −0.6767. Protein content (0.8710), lipid content (0.7988), tannin content (0.4783), and amylose content (0.2747) loaded positively on the quality-indicator construct, while amylopectin content (−0.8592) and total starch content (−0.8537) showed negative loadings. Grain yield was represented by a loading coefficient of 1.0.

## 4 Discussion

### 4.1 Divergence among production bases in soil properties and sorghum grain composition

Among the three sampled production bases, we observed substantial heterogeneity in soil nutrient status and grain nutritional profiles, consistent with previous investigations in sorghum-producing areas of southwest China (Xie et al., 2024). Renhuai (RH) harbored the highest soil organic matter (SOM) and total phosphorus (TP) contents, which corresponded to the highest total starch and amylopectin concentrations in sorghum grains. This positive association between soil phosphorus availability and starch accumulation has been well documented in cereal crops (Osman et al., 2022), as phosphorus is a core component of energy carriers (ATP) and sugar phosphate intermediates that directly fuel starch biosynthesis in developing endosperms. In contrast, Bijie (BJ), with the highest total potassium (TK) but lowest SOM, exhibited the lowest total starch content but elevated lipid and protein levels. This pattern aligns with the established understanding that potassium regulates protein synthesis and lipid metabolism in grains, while low soil carbon availability may redirect plant carbon allocation toward structural and nitrogenous storage compounds rather than starch (Osman et al., 2022).

Notably, Jinsha (JS) achieved the highest grain yield despite having intermediate soil nutrient levels, suggesting that yield variation among the sampled production bases may reflect the combined influences of environmental conditions, agronomic management, and genotype rather than soil nutrient status alone (Ostmeyer et al., 2022). The lower tannin content in JS grains also carries important implications for liquor-making quality: moderate tannin levels contribute to flavor complexity during fermentation, whereas excessive tannins reduce starch digestibility and may impart undesirable astringency (Xie et al., 2024). Compared with a previous regional survey in Guizhou sorghum zones (Xie et al., 2024), our study observed a slightly different ranking of total starch among the sampled bases, which may be attributed to differences in sampling years, specific cultivar distributions, and fertilization regimes at the sampled field sites.

### 4.2 Biogeographic patterns of AMF communities at the genus level

At the genus level, Paraglomus, Glomus, and unclassified taxa collectively dominated the AMF communities across all samples, a pattern partially consistent with previous findings in agricultural and karst ecosystems (You et al., 2022; Zhang et al., 2025). However, unlike many studies that report Glomus as the absolute dominant genus in conventional farmland soils (You et al., 2022), Paraglomus exhibited the highest relative abundance among classified genera in most samples, particularly in the Bijie site. The predominance of Paraglomus in high-altitude, potassium-rich karst soils may reflect its unique adaptive strategies: species of Paraglomus often possess strong stress tolerance and efficient potassium mobilization capacity, enabling them to thrive in nutrient-imbalanced calcareous soils with high calcium and potassium contents (Yan et al., 2023).

The remarkably high proportion of unclassified AMF taxa in Renhuai samples indicates that karst agroecosystems may harbor a large reservoir of novel or under-documented AMF lineages, which is congruent with the high microbial endemism reported in southwest China’s karst regions (You et al., 2022). The LEfSe analysis identified Glomeromycetes as a differentially represented taxon for the RH base, whereas Paraglomeraceae and Paraglomeromycetes were enriched in Bijie. This taxonomic differentiation at higher taxonomic ranks is consistent with long-term environmental filtering shaping the phylogenetic structure of AMF communities among the sampled bases, rather than merely causing species-level shifts (Ma et al., 2022). These taxonomic differences represent contrasts among the three sampled bases; whether similar patterns hold across other production sites in the three regions remains to be tested.

### 4.3 Decoupled alpha and beta diversity patterns of AMF communities

An intriguing finding of this study is that alpha diversity indices (Shannon, Simpson, Pielou evenness, and dominance) showed no significant differences among the three sampled production bases, whereas PCoA and MRPP consistently revealed highly significant separation in community composition (beta diversity). This decoupled pattern suggests that the three sampled bases harbor AMF communities with similar species richness and evenness but distinct species identities, reflecting strong species turnover along environmental gradients rather than changes in overall community complexity (Yan et al., 2023). Such a biogeographic pattern is commonly observed in soil microbial communities at regional scales, where local environmental conditions select for different species from the regional pool while maintaining comparable within-site community complexity (Wei et al., 2025).

The broader dispersion of Bijie samples in the ordination space compared with Renhuai and Jinsha indicates higher within-base heterogeneity in AMF communities, which is likely associated with the more variable topography and fragmented microhabitats in the high-altitude Bijie area (Yan et al., 2023). Previous studies along elevation gradients have also reported that AMF community heterogeneity increases at higher elevations due to discontinuous habitats and stronger environmental filtering at small spatial scales (Shulman et al., 2026).

### 4.4 Environmental correlates of AMF community assembly

The CCA ordination clearly separated AMF communities along axes corresponding to soil nutrient gradients and climatic-topographic gradients, showing that AMF community composition covaried with both edaphic and climatic gradients in sorghum fields (Ma et al., 2022; Ndeko et al., 2024). Specifically, SOM and average monthly temperature were strongly associated with the Renhuai AMF community. Soil organic matter not only provides carbon substrates for AMF spore germination and extraradical hyphal growth but also modulates soil physical structure and nutrient bioavailability, and may therefore influence AMF community composition (Etesami et al., 2021). Meanwhile, temperature is a well-recognized correlate of AMF biogeography, as different AMF taxa have distinct thermal optima for growth and symbiotic functioning (Ma et al., 2022).

By comparison, the Bijie samples were positioned along a broader environmental gradient characterized by higher TK, elevation, AWS, and NSR. Because these variables showed strong multicollinearity, their individual contributions cannot be separated in the present dataset and should therefore be interpreted collectively rather than as independent environmental effects. Elevation per se does not directly affect AMF but acts as a comprehensive proxy for temperature, precipitation, and soil development gradients (Yan et al., 2023). The TK-associated gradient is noteworthy, as potassium has been less frequently reported as a key correlate compared with nitrogen and phosphorus; however, in potassium-rich karst soils, potassium availability may represent a selective filter favoring taxa with efficient potassium transport and exchange capabilities, such as *Paraglomus* and *Gigaspora* (Masebo et al., 2023). Because environmental variables were characterized at the base level and several were strongly collinear, the present results identify site-associated environmental gradients but cannot attribute community variation to any single regional factor.

### 4.5 Functional implications of AMF communities and the soil–microbe–crop cascade

FUNGuild annotation of the independent ITS1 dataset indicated differences in the predicted trophic-mode composition of the broader fungal community among the sampled production bases (Supplementary Figure S2). In particular, Bijie samples showed relatively high proportions of taxa assigned to the symbiotroph trophic mode. However, the FUNGuild “symbiotroph” category encompasses multiple forms of fungal symbiosis and is not specific to AMF (Frew, 2025). Therefore, this pattern should be interpreted only as a difference in the predicted trophic organization of the broader rhizosphere fungal community and not as evidence of greater AMF abundance, activity, or host mycorrhizal dependency. Further taxon-specific or functional measurements would be required to determine the ecological significance of this pattern (Elsafy et al., 2025).

The PLS path model provided quantitative insights into the multivariate relationships among climate, AMF, soil properties, and crop performance. The strongest positive path was observed from soil properties to grain quality indicators, highlighting the close association between edaphic conditions and sorghum nutritional quality (Xie et al., 2024; Osman et al., 2022). Interestingly, soil properties showed a significant negative association with grain yield, which may reflect the well-documented yield–quality trade-off in cereal crops: high soil fertility, particularly high nitrogen availability, often promotes vegetative growth and yield formation but dilutes grain protein and starch concentrations (Osman et al., 2022). The core AMF community exhibited a significant positive association with soil properties, indicating that favorable soil nutrient status supports a more structured and functionally competent AMF community (Etesami et al., 2021). Although the direct paths from AMF to yield and quality did not reach statistical significance, the positive coefficient from AMF to quality indicator suggests a potential association between AMF community composition and grain nutritional quality, which is supported by inoculation experiments showing that AMF can enhance protein and mineral contents in sorghum grains (Elsafy et al., 2025; Gill et al., 2025).

### 4.6 Limitation

Several limitations should be considered when interpreting these results. First, only one production base was sampled within each geographic region, and climate data were obtained from one weather station per region. Consequently, site, base, and region are completely confounded in the present design, and the study provides no independent replication of the regional factor. All reported differences should therefore be interpreted strictly as contrasts among the three sampled bases and cannot be generalized to other sorghum fields in Renhuai, Jinsha, or Bijie. Second, several climatic variables were characterized at the production-base level and consequently covaried with geographic location, preventing their independent effects from being separated statistically. Third, the relatively small number of field sampling units (*n* = 12) limits the complexity and stability of multivariate models such as CCA and PLS-PM. The path model in particular should be regarded as hypothesis-generating rather than confirmatory. Fourth, the observational design precludes causal inference. All reported associations between environmental variables, AMF communities, and crop variables are correlational. Future studies should incorporate multiple independent fields within each region, subplot-level environmental measurements, larger sample sizes, and controlled manipulative experiments to test the ecological mechanisms suggested by the present site-level associations.

## 5 Conclusion

In summary, the three sampled Jiang-flavor baijiu sorghum production bases differed in soil properties, grain characteristics, and rhizosphere AMF community composition. Although alpha-diversity indices were broadly comparable, community composition showed clear site-associated variation, with Paraglomus representing the predominant classified genus in most samples. Environmental ordination suggested that variation in AMF community composition was associated with average monthly temperature, precipitation, average sunshine hours, slope, soil organic matter, and total phosphorus. However, because several variables were strongly collinear and measured at the base level, these relationships were interpreted collectively rather than as independent effects.

Given that only one production base and one weather station were sampled per region and that the study was observational in design, the present findings should not be generalized beyond the three sampled sites or interpreted as evidence of causal environmental or microbial effects. The results provide a site-level baseline for AMF community variation among the three sampled bases and support future studies using replicated fields and manipulative experiments to determine how soil conditions and AMF communities interact with sorghum performance.

## Data Availability

The datasets presented in this study can be found in online repositories. The names of the repository/repositories and accession number(s) can be found at: https://www.ncbi.nlm.nih.gov/, PRJNA1497899.

## Footnotes

1
https://data.cma.cn/
