# Supplementary material for: Arbuscular mycorrhizal fungal communities in Jiang-flavor baijiu sorghum rhizosphere: environmental correlates and variation among production bases
Source: Front Microbiol. 2026 Oct 29;17:1965730. doi: 10.3389/fmicb.2026.1965730 (PMC13754833; doi:10.3389/fmicb.2026.1965730)
Supplement: Supplementary file 1 [file Supplementary_file_1.DOCX]

Table S1. Comparison of Soil Carbon, Nitrogen, Phosphorus, and Organic Matter Contents Across Different Regions

| The sampling area | SOM/·kg^-1^ | TN/g·kg^-1^ | TP/g·kg^-1^ | TK/g·kg^-1^ |
| --- | --- | --- | --- | --- |
| Renhuai | 18.32 | 2.63±0.02 | 1.98±0.01 | 11.88 |
| Jinsha | 16.77 | 2.75±0.01 | 1.64±0.02 | 13.12 |
| Bijie | 16.21 | 2.44±0.02 | 1.88±0.04 | 14.56 |

Table S2. Grain yield across different regions

| Regions | RH | JS | BJ |
| --- | --- | --- | --- |
| Grain yield （kg ha^-1^） | 1650±47 b | 1977±26 a | 1432±26 c |

Note: Values are presented as mean ± standard error. Different lowercase letters in the same row indicate significant differences in grain yield among regions at the *p* < 0.05 level.

Table S3. Stepwise multicollinearity screening of environmental variables prior to canonical correspondence analysis (CCA)

| Variable retained for CCA | Final VIF |
| --- | --- |
| AMT | 1.33 |
| Pr | 1.75 |
| ASH | 6.81 |
| Slo | 1.14 |
| SOM | 6.83 |
| TP | 2.52 |

Abbreviations: AMT, average monthly temperature; Pr, precipitation; RH, relative humidity; AWS, average wind speed; NSR, net solar radiation; ASH, average sunshine hours; ELE, elevation; Slo, slope; SOM, soil organic matter; TP, total phosphorus; TN, total nitrogen; TK, total potassium. VIF, variance inflation factor.

Figure S1. Shannon‑Wiener diversity rarefaction curves of all samples.

Note: Rarefaction curves based on Shannon index reflecting microbial community diversity as a function of sequencing depth. The x‑axis represents the number of sampled sequences, and the y‑axis represents the Shannon‑Wiener diversity index. Each curve corresponds to an individual sample (A1‑A4, B1‑B4, C1‑C4). All curves gradually plateaued, indicating that the sequencing depth was sufficient to capture the majority of microbial diversity in the present samples.

Figure S2. Relative abundance of FUNGuild-predicted trophic modes (symbiotroph vs. unassigned) in rhizosphere soil based on ITS1 sequencing. Renhuai samples (A1–A4), Jinsha samples (B1–B4), and Bijie samples (C1–C4) are shown. Note: This analysis is based on the total fungal ITS1 dataset, not AMF-targeted 18S rRNA gene data.
